# Structural Basis of Binding and Rationale for the Potent Urease Inhibitory Activity of Biscoumarins

**DOI:** 10.1155/2014/935039

**Published:** 2014-09-09

**Authors:** Muhammad Arif Lodhi, Sulaiman Shams, Muhammad Iqbal Choudhary, Atif Lodhi, Zaheer Ul-Haq, Saima Jalil, Sarfraz Ahmad Nawaz, Khalid Mohammed Khan, Sajid Iqbal, Atta-ur Rahman

**Affiliations:** ^1^Department of Biochemistry, Abdul Wali Khan University, Mardan, Khyber Pakhtunkhwa 23200, Pakistan; ^2^Dr. Panjwani Center for Molecular Medicine and Drug Research, International Center for Chemical and Biological Sciences, University of Karachi, Karachi 75270, Pakistan; ^3^H. E. J. Research Institute of Chemistry, International Center for Chemical and Biological Sciences, University of Karachi, Karachi 75270, Pakistan; ^4^Departament de Didàctica de les Ciències Experimentals i la Matemàtica, Universitat de Barcelona, Spain; ^5^Institute of Chemistry, University of the Punjab, Quaid-i-Azam Campus, Lahore 54590, Pakistan

## Abstract

Urease belongs to a family of highly conserved urea-hydrolyzing enzymes. A common feature of these enzymes is the presence of two Lewis acid nickel ions and reactive cysteine residue in the active sites. In the current study we examined a series of biscoumarins **1**–**10** for their mechanisms of inhibition with the nickel containing active sites of Jack bean and *Bacillus pasteurii* ureases. All these compounds competitively inhibited Jack bean urease through interaction with the nickel metallocentre, as deduced from Michaelis-Menten kinetics, UV-visible absorbance spectroscopic, and molecular docking simulation studies. Some of the compounds behaved differently in case of *Bacillus pasteurii* urease. We conducted the enzyme kinetics, UV-visible spectroscopy, and molecular docking results in terms of the known protein structure of the enzyme. We also evaluated possible molecular interpretations for the site of biscoumarins binding and found that phenyl ring is the major active pharmacophore. The excellent in vitro potency and selectivity profile of the several compounds described combined with their nontoxicity against the human cells and plants suggest that these compounds may represent a viable lead series for the treatment of urease associated problems.

## 1. Introduction

Urease (urea amidohydrolase, EC: 3.5.1.5) occurs throughout the animal and plant kingdom; many microorganisms use this enzyme to provide a source of nitrogen for growth, and it also plays an important role in plant nitrogen metabolism during the germination process [1, 2]. The presence of urease activity in soils is exploited in the widespread agricultural practice of urea-based fertilizer application for enhancing crop yields. Unfortunately, excessive levels of soil urease can degrade fertilizer's urea too rapidly and result in phytopathic effects and loss of volatilized ammonia [3]. On the other hand, in medical and veterinary science, urease has been identified as a virulence factor in certain human and animal pathogens; it participates in the development of kidney stones, pyelonephritis, peptic ulcers, and other disease states [4]. The obvious remedy for treating bacterial infection is antimicrobials, however, this has often proven futile [5], and only a few combination regiments have reached to clinical practice. Thus, the need for alternative or novel treatment is greatly felt. The discovery of potent and safe urease inhibitors have been an important area of pharmaceutical research due to the involvement of ureases in different pathological conditions. We have previously reported a number of novel synthetic and natural inhibitors of urease and their inhibition kinetics and structure-activity relationship studies [6–9]. In continuation of our efforts to discover new and potent inhibitors of medicinally important enzymes through high-throughput screening assays, we identified these biscoumarins, having appreciable efficacy against ureases.

The objective of the current investigation was to explore the possible binding interactions of biscoumarin compounds in the target protein. These structural studies may guide future drug design to improve the selectivity and efficacy by introducing appropriate substituents on the biscoumarin molecular scaffold for the rational design of new chemical inhibitory molecules. We have selected biscoumarin class of compounds for this study because this class has never been studied for their binding interpretations before and we are presenting, for the first time, the mechanism of binding of** 1**–**10** in urease enzyme.

## 2. Materials and Methods

### 2.1. Urease Assay and Inhibition

Reaction mixtures comprising 25 *μ*L of enzyme (Jack bean and* Bacillus pasteurii* ureases) solution and 55 *μ*L of buffers containing urea (2–24 mM for jack bean and* Bacillus pasteurii* ureases) were incubated with 5 *μ*L of test compounds at 30°C for 4.15 min in 96-well plates. The increasing absorbance at 560 nm was measured after 10 min, using a microplate reader (Molecular Devices, USA). All reactions were performed in triplicate in a final volume of 200 *μ*L. The results (change in absorbance per min.) were processed by using SoftMax Pro software (Molecular Devices, USA). All the assays were performed at pH 6.8 (3 mM sodium phosphate buffer) and 7 *μ*g of phenol red per mL as indicator [10]. Percentage inhibitions were calculated from the formula 100 − (OD_testwell_/OD_control_) × 100. Thiourea was used as the standard inhibitor of urease.

#### 2.1.1. Determination of Kinetic Parameters

The concentration of tested compounds that inhibited the hydrolysis of substrates (Jack bean urease and* Bacillus pasteurii* ureases) by 50% (IC_50_) was determined by monitoring the effect of various concentrations of the compounds in the assays on the inhibition values. The IC_50_ (inhibitor conc. that inhibits 50% activity of both enzymes) values were then calculated using the EZ-Fit Enzyme Kinetics program (*Perrella Scientific Inc., Amherst*, USA).

**Scheme 1:**
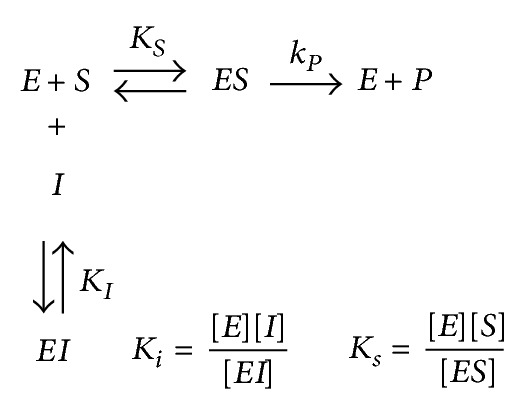
Competitive inhibition.

**Scheme 2:**
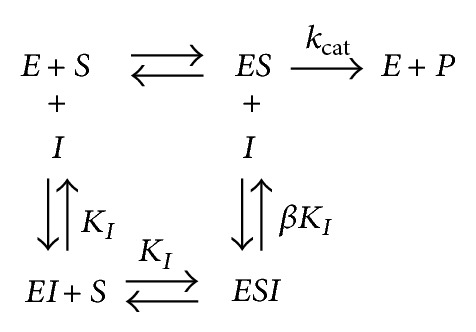
Noncompetitive inhibition.

**Scheme 3:**
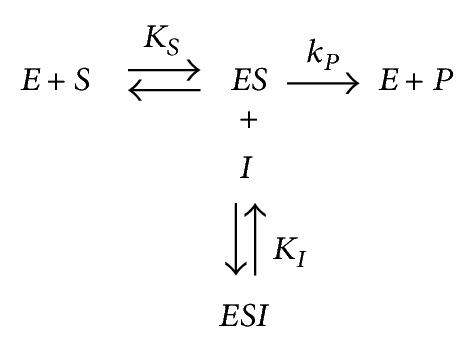
Uncompetitive inhibition.

The interaction of** 1**–**10** with Jack bean (J.B.) urease and* Bacillus pasteurii *urease (B.P.) are presented by Schemes 1, 2, and 3.

Where* ES* is the J.B. urease-urea or B.P. urease-urea complex and *P* is the product. *K*_1_ and *βK*_1_ are the inhibition constants reflecting the interactions of inhibitors with the free J.B. urease or B.P. urease and the J.B. urease-urea or B.P. urease-urea complexes, respectively.

Dissociation constant/inhibition constant (*K*_*i*_) were determined by the interpretation of Dixon plot [11]. Lineweaver-Burk plot [12] and their secondary replots using initial velocities obtained over a substrate concentration range between 2.0 and 24 mM urea for J.B. and B.P. ureases, respectively. Nonlinear regression equations were used to determine the values of *K*_*I*_, *K*_*m*_, and *V*_max⁡_ in the Lineweaver-Burk plot and Dixon plots. The *K*_*i*_ values dissociation constant/inhibition constant of J.B. urease-inhibitor or B.P. urease-inhibitor complex into free J.B. urease or B.P. urease and inhibitor was determined graphically by Dixon plot and Lineweaver-Burk plots.

#### 2.1.2. Statistical Analysis

Graphs were plotted using GraFit program [13]. Values of the correlation coefficients, slopes, intercepts, and their standard errors were obtained by the linear regression analysis using the same program. The correlation for all the lines of all graphs was found >0.99. Each point in the constructed graphs represents the mean of three experiments.

### 2.2. Molecular Docking Simulations

**Figure 1:**
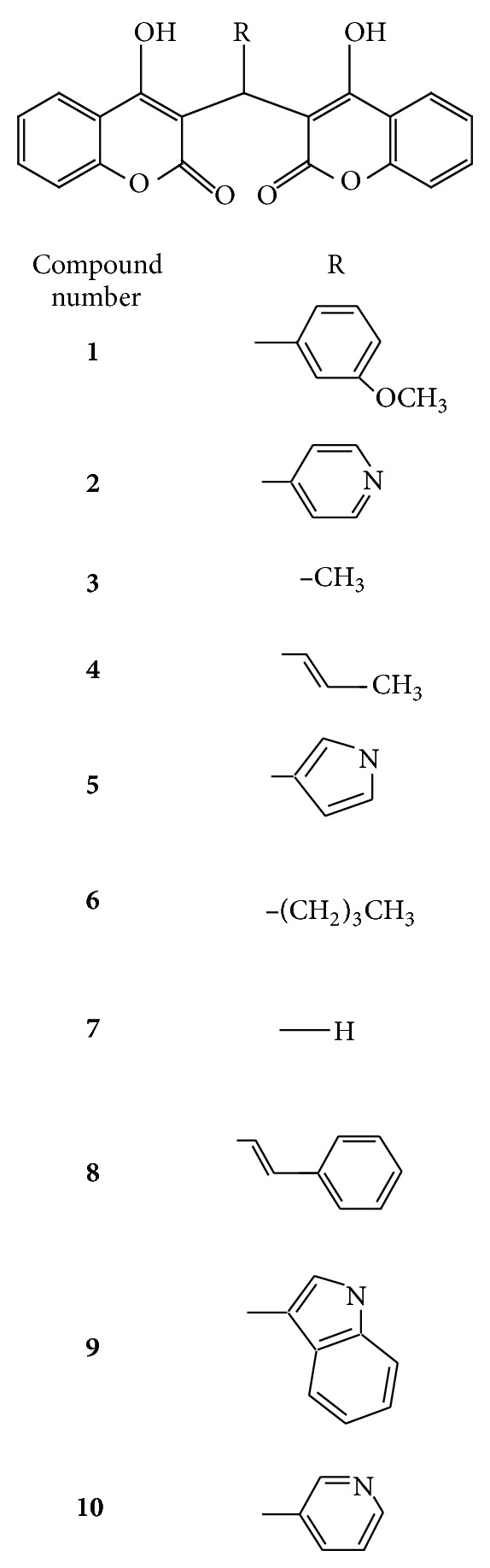
Chemical structures of biscoumarin** 1**–**10**.

The accurate prediction of protein ligand interaction geometries is important for the success of structure-based drug design. It requires docking tools that are able to generate suitable configurations and conformations of a ligand within a protein binding site and scoring functions that appropriately translate interaction geometries into an energetic measure describing the quality of the interaction. In our present study molecular docking study was conducted by using FlexX. The three-dimensional structures of biscoumarins were constructed using the SYBYL program (Figure 1) [14]. The docking studies were carried out using FlexX [15] docking software. For FlexX energy minimization was performed using the tripos force field with a distance gradient algorithm with convergence criterion of 0.05 KCal/(mol Å) and maximum 1000 interactions, respectively. FlexX software is a fast and flexible algorithm for docking small ligands in binding sites of the enzymes, using an incremental construction algorithm that actually builds the ligands in the binding site [14]. The software incorporates protein-ligand interactions, placement of the ligand core, and rebuilding the complete ligand. As docking algorithm a Monte Carlo simulated annealing search process was used starting at a temperature corresponding to RT = 1200 cal/mol, which was reduced by a factor of 0.90 after each cycle. A cycle consisted of a maximum of 30,000 accepted or rejected steps, where a step corresponds to a random change in translational, rotational, and torsional degrees of freedom of the ligand. One hundred cycles were performed per docking experiment, and for each ligand 100 independent dockings were carried out. The charges of the ligands were obtained using the standard RESP procedure [15]. The necessary* ab initio* calculations were performed with GAUSSIAN98 [15]. Docking results were analyzed by VMD [16] and LIGPLOT [17].

### 2.3. Cytotoxic Evaluation

#### 2.3.1. Viability of Human Neutrophils

Heparinized fresh venous blood was drawn from healthy volunteers in a local blood bank and the neutrophils were isolated by developed method of McLaughlin et al. [18, 19]. 45 The whole blood was mixed with Ficoll-Paque (Pharmacia Biotech Amersham, Uppsala). After sedimentation the unwanted red blood cells (RBCs) were layered as a buffy coat on the cushion of Ficoll (3.0 mL). It was then centrifuged for 30 min. at 1500 rpm, and supernatant was discarded. Further treated with hypotonic ammonium chloride solution (0.83%) to lyse RBCs. The preparation was then centrifuged and the neutrophils were washed with Modified Hank's Solution (MHS) and resuspended at a concentration of 1 × 10^7^ cells/mL.

#### 2.3.2. Assay Procedure

Isolated human neutrophils (1 × 10^7^ cells/mL) were incubated first with test compounds for 30 min., and then after the addition of 0.25 mM WST-1 (Dojindo Laboratories Kumamoto, Japan) in water bath shaker at 37°C [18, 19]. After 3 hrs incubation, change in the absorbance was measured at 450 nm in 96-well plate by using microplate reader Spectra MAX 340 (Molecular Devices, CA, USA). The OD is the mean of the five experimental replicates. The percentage (%) cell viability was calculated by using the following formula:

(1) Percentage  (%)  viability  of  cells  ={(OD  test  compound×100OD  control)−100}−100.

### 2.4. Spectral Interaction Studies of** Biscoumarins **with Urease

In the absence of any ligand, urease displays an absorption spectrum, based on *Mr* = 590,000 and 746.4, 523.6, 386.8, and 313.2 nm (*ε*° = 2,300 M^−1** **^cm^−1^, based on *Mr* = 230,000). This spectrum is essentially pH-independent. Binding of biscoumarin enhances the absorbance in three regions between 800 and 200 nm with the exact wavelength depending upon the biscoumarin structure. For example, the difference spectra maxima were 342, 390, and 286 nm for compound** 7** (Figure 11), versus 389, 341, and 523 nm for compound** 2**. These spectral interactions are similar to those with biscoumarin-nickel complexes in case of compound 7 and same happened with all competitive inhibitors. A double-reciprocal plot (Figure 12) of the absorbance changes versus biscoumarin concentration provided the binding constant (*K*_*d*_ = 16.50 *μ*M for compound** 7**). The spectrally determined *K*_*d*_ value is nearly identical to the *K*_*i*_ determined kinetically for compound** 7**.

### 2.5. Assay for Chelating Property of the Selected Urease Inhibitors

Allligands were added at concentration equal to their *K*_*i*_ to 0.1 M of NiCl_2_ and their possible chelating effects were compared to those of 25 *μ*M EDTA, by following absorbance spectrum of the NiCl_2_ from 190 to 900 nm and results are shown in Figures 9 and 10.

### 2.6. Phytotoxicity Evaluation Protocol

The assay was performed according to the modified protocol of Bremner [20]. The test compounds were dissolved in sterilized E-medium at different concentrations, that is, 5, 50, 500 *μ*g/mL in methanol. Sterilized conical flasks were inoculated with compounds with desired concentrations prepared from the stock solution and allowed to evaporate overnight. Each flask was inoculated with 20 mL of sterilized E-medium and ten* Lemna aequinoctialis* Welv. each containing a rosette of three fronds. Other flasks were supplemented with methanol serving as negative control and reference inhibitor, that is, phosphoroamide (a urease inhibitor already in commercial use) serving as positive control. The treatments were replicated three times and the flasks incubated at 30°C Fisons Fi-Totron 600 H growth cabinet for seven days, 9000 lux light intensity, 56 ± 10 rh (relative humidity), and 12 h day length. Growth of* Lemna aequinocitalis* in compound containing flask was determined by counting the number of fronds per dose and growth inhibition calculated with reference to negative control.

## 3. Results and Discussion

Urease is an enzyme that is present in many plants and soil. It catalyzes the hydrolysis of urea to ammonium and carbamate ions, which are decomposed to carbon dioxide and ammonia. The active site contains two nickel (II) atoms which, as shown by X-ray analysis, are linked by a carbamate bridge, furthermore, two imidazole nitrogen atoms are bound to each nickel atom, and a carboxylate group and a water molecule fill the remaining coordination site of the metal ion [4]. The coordination geometry of the first nickel atom is pseudo tetrahedral, while that of second is roughly trigonal bipyramidal. In order to discriminate among the inhibition capacities of various compounds, it is important to understand the coordination mechanism between the active site of the enzyme and the inhibitor. In jack bean urease, one nickel is suggested to coordinate a water molecule, and a second nickel coordinates hydroxide. Urea displaces the water molecule and the illustrated resonance structure is thought to coordinate to nickel such that the positively charged nitrogen is electrostatically stabilized by a nearby carboxylate anion. A general base is proposed to activate the nickel-coordinated hydroxyl group for nucleophilic attack on the urea carbon to form a tetrahedral intermediate. Decomposition of this intermediate and release of ammonia is thought to include general acid catalysis by a nearby thiol group. Finally, carbamate is released with regeneration of enzyme [4]. The biscoumarins described in the current investigation have a basic coumarine skeleton, which differs by substitution at “**R**” position. This study has resolved the inhibitory mechanism of these compounds that are competitive inhibitors of jack bean urease. In case of B.P. urease the compounds** 1**,** 6**,** 7**,** 8**, and** 10** inhibit the enzyme competitively while** 3**,** 4**,** 5**, and** 9** inhibit it through uncompetitive mechanism, whereas compound** 2** perturbs the enzyme activity in noncompetitive manner.

**Figure 2:**
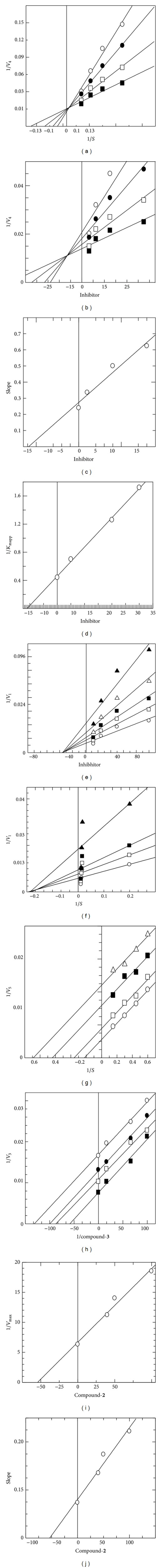
Steady state inhibition of J.B. urease by compound** 7**; (a) is the Lineweaver-Burk plot of reciprocal of initial velocities versus reciprocal of four fixed urease concentrations in absence (■) and presence of 12.5 *µ*M (□), 25.0 *µ*M (●), and 50 *µ*M (○) of compound** 7**. (b) is the Dixon plot of reciprocal of the initial velocities versus various concentrations of compound** 7** at fixed urease concentrations, (■) 50 *µ*M, (□) 25.0 *µ*M, (●) 12.5 *µ*M, and (○) 6.2 *µ*M; (c) is the, 1/*K*_*m*app_ versus various concentrations of compound** 1**. (d) is the, 1/Slope versus various concentrations of compound** 7**. (e) is the Lineweaver-Burk plot of reciprocal of initial velocities versus reciprocal of four fixed B.P. urease concentrations in absence (□) and presence of 12.5 *µ*M (□), 25.0 *µ*M (■), and 50 *µ*M (▼) of compound** 2**. (f) is the Dixon plot of reciprocal of the initial velocities versus various concentrations of compound** 2** at fixed urease concentrations, (○) 50 *µ*M, (□) 25.0 *µ*M, (■) 12.5 *µ*M, and (▼) 6.2 *µ*M. (g) is the 1/*K*_*m*app_ versus various concentrations of compound** 2**. (h) is the, 1/Slope versus various concentrations of compound** 2**. (i) is the Lineweaver-Burk plot of reciprocal of initial velocities versus reciprocal of four fixed B.P. urease concentrations in absence (○) and presence of 12.5 *µ*M (□), 25.0 *µ*M (■), and 50 *µ*M (∆) of compound** 3**. (j) is the Dixon plot of reciprocal of the initial velocities versus various concentrations of compound** 3** at fixed urease concentrations, (■) 50 *µ*M, (□) 25.0 *µ*M, (▼) 12.5 *µ*M, (▼) 6.2 *µ*M, and (○) 3.1 *µ*M.** K** is the 1/*K*_*m*app_ versus various concentrations of compound** 3**.** l** is the 1/Slope versus various concentrations of compound** 3**.

The enzyme kinetic studies have revealed that compounds** 1**–**10** inhibited both the ureases in a concentration-dependent manner with *K*_*i*_ values ranging between 15.0–75.0 *μ*M and 13.3–68.1 *μ*M against J.B. and* Bacillus* B.P. urease, respectively. *K*_*i*_ values were calculated in three different ways; firstly, the slopes of each line in the Lineweaver-Burk plot were plotted against different concentrations of inhibitors; secondly the 1/*V*_maxapp_ was calculated by plotting different fixed concentrations of J.B. and B.P. urease versus Δ*V* in presence of different fixed concentrations of inhibitors in the respective assays for both the ureases. The *K*_*i*_ was then calculated by plotting different concentrations of inhibitor versus 1/*V*_maxapp_ which was the intercept on the *x*-axis. Thirdly, *K*_*i*_ was directly measured from Dixon plot as an intercept on *x*-axis. Determination of the type of inhibition is critical for identification of the mechanism of inhibition as well binding sites of the inhibitors. Lineweaver-Burk, Dixon plots, and their replots indicated that all compounds except** 3**,** 4**,** 5**, and** 9** exhibited pure uncompetitive type of inhibition against B.P. urease with compound** 2** exhibiting noncompetitive inhibition, while, all the rest of the compounds inhibited both the enzymes competitively, as in these cases there was increase in *K*_*m*_ values without affecting the *V*_max⁡_ of the enzymes. In other words our findings clearly show that the inhibitor binds specifically at the active site in most of the tested compounds. On the other hand** 3**,** 4**,** 5**, and** 9** displayed uncompetitive type of inhibition against B.P. urease because in these cases there were decrease in both *V*_max⁡_ and *K*_*m*_ values. The steady-state kinetics analysis of B.P. urease inhibition by compounds** 2**,** 3**,** 4**,** 5**, and** 9** produces parallel lines seen in double reciprocal plot suggested either of two interpretations. (1) Classical uncompetitive inhibition occurs when an inhibitor binds to an enzyme-substrate complex. This type of inhibition is typically observed with multisubstrate enzymes; however, uncompetitive could reasonably interact with hydrolytic enzyme at side normally occupied by catalytic water. (2) Parallel double-reciprocal plots may also arise for inhibitors that bind to a form of the enzyme (E*) generated from the active enzyme species (E) during the catalysis. The *K*_*i*_, *K*_*m*_, *V*_max⁡_, *V*_maxapp_, and *K*_*m*app_ values and their type of inhibition are listed in Table 1. The graphical analysis of steady-state inhibition for** 7**,** 2**, and** 3** against J.B. urease and B.P. urease is shown in Figure 2.

The accurate prediction of protein-ligand interaction geometries is essential for the success of structure-based drug design. It requires docking tools that are able to generate suitable configurations and conformations of a ligand within a protein binding site and scoring functions that appropriately translate interaction geometries into an energetic measure describing the quality of the interaction. In order to understand the in-depth mechanism of inhibition the compound** 1**–**10** were subjected to molecular docking simulation and UV-visible spectroscopic studies. Docking position with the lowest energy was found most often during the docking procedure. This indicates that the phase space is sufficiently sampled; also we repeated the docking protocol for each compound many times and found that best docking positions and their respective minimum energies are consistently reproduced. The size and shape of our compounds support a Ni metalocentre spanning binding mode in most of the cases. Therefore urease cocrystallized with Acetohydroxamic acid (4UBP) [1], was taken for the comparison in order to control the performance of our docking approach. The best ranked docking solutions showed that all the tested ligands could accommodate ideally inside the nickel metallocentre, except** 2**,** 3**,** 4**,** 5**, and** 9** that were found to interact outside the Ni metal centre. Generally speaking in the present study, the compounds having bulky and nonplanar moieties attached at (R) position were found to be relatively less potent inhibitors of both ureases. Moreover, all the tested compounds were potent inhibitors of B.P. urease as compared J.B. urease except the compound** 5** which was selective for J.B. uraese.

It was observed further that the inhibitory activities of biscoumarins were dependent on the length of preincubation with the urease. It indicates that prolonged interaction between the ureases and the inhibitors is needed in order to make a stable enzyme inhibitor complex, as shown in Figure 13.

### 3.1. Compound **7**-Urease Complex

**Figure 3:**
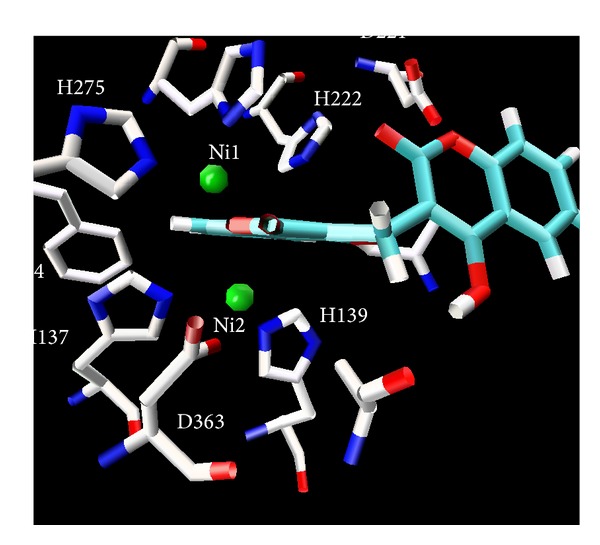
Ligand** 7** in the active site of urease, showing that phenyl ring of the ligand is symmetrically placed inside the active site urease (*Bacillus pasteurii*).

**Figure 4:**
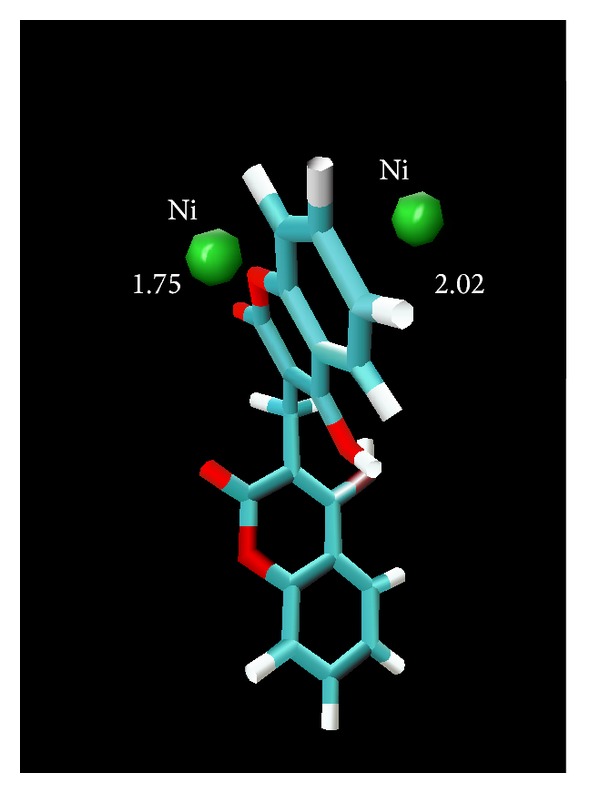
A view showing that the benzene ring of ligand** 7** is sandwiched between two Nickel ions in active site of urease (*Bacillus pasteurii*), showing that phenyl ring of the ligand is symmetrically placed inside the active site.

**Figure 5:**
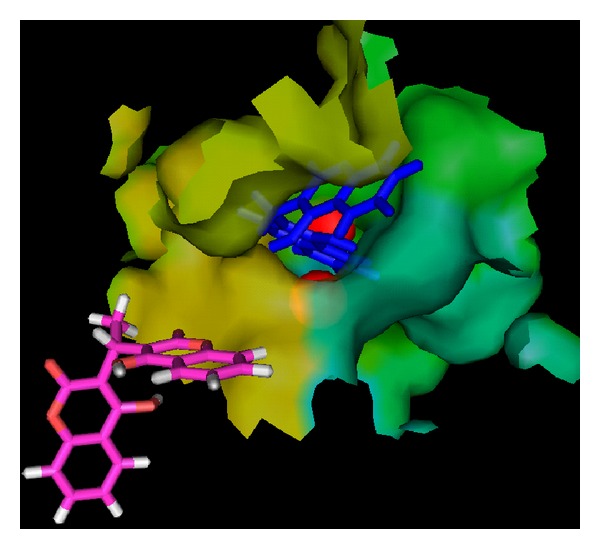
Showing the interaction of compound** 7 **(blue) and** 3 **(red) with urease (*Bacillus pasteurii*). Ligand** 7** is penetrated inside the active site and interacted with nickel metallocentre of urease but ligand** 3 **is unable to reach the narrow pocket.

**Figure 6:**
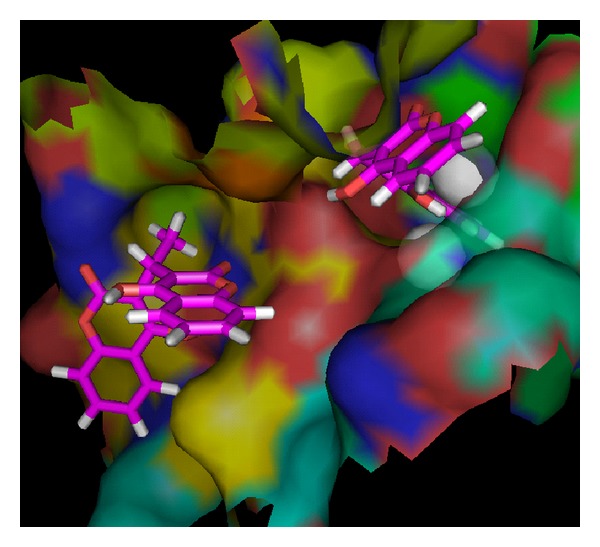
Another view showing the interaction of compounds** 7** and** 3** with urease (*Bacillus pasteurii*) from another angle. Ligand** 7** is penetrated in to the active site but ligand** 3** is unable to reach probably because of its shape, the phenyl ring of the ligand** 7** is symmetrically placed inside the active site.

**Figure 7:**
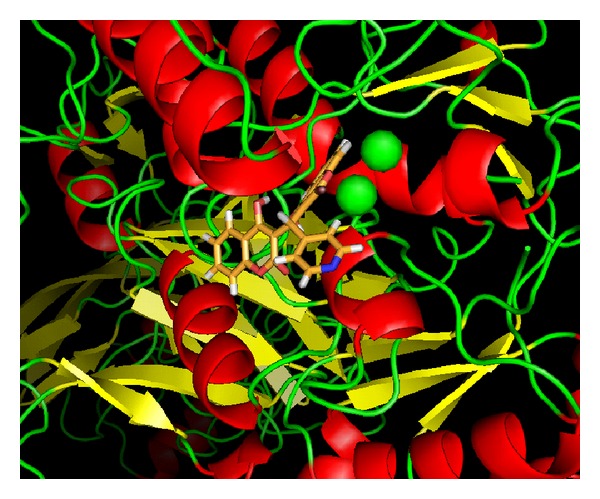
A view showing the interaction of compound** 2 **(yellow) with urease (*Bacillus pasteurii*). Ligand** 2** is unable to reach the nickel (green) metalocentre and bind outside the active site. Helix (Red), sheet (yellow), and loop (green).

**Figure 8:**
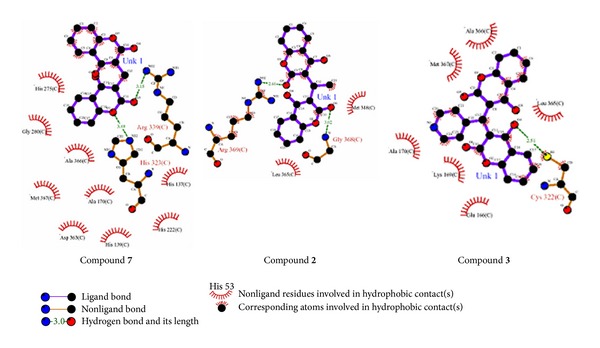
2D-Schematic representation of compounds** 7**,** 2,** and** 3** by LIGPLOTS, showing that hydrophobic contacts and hydrogen bonding are the other interactions, holding the ligand-receptor complexes in stable form.

The complex of compound-**7** with urease is stabilized mainly through chelation of phenyl ring which is symmetrically placed between positively charged Ni ions in the active site and hindered the entry of substrate. The demonstration of nickel-Phenyl interactions using a competitive inhibitor, where the spectrally observed *K*_*d*_ is identical to the *K*_*i*_, provides strong support for a mechanism where urea also binds to the nickel metallocenter while UV-visible spectroscopic studies also gives us strong evidence and we observed very sharp increase in absorbance in the 286 nm region which is of course for coordinated benzene ring of biscoumarin with the nickel of urease (results are shown in Figure 11). The further stability is given by hydrogen-bonding interaction of the Arg 339 (3.15 Å) and His 323 (3.09 Å) of the active centre. Hydrophobic contacts with the residue (His 275, Gly 280 Ala 366, Met 367, Ala 170, His 137, Asp 363, His 139, and His 222) are also responsible for potent competitive type of inhibition of this compound. All other ligands which inhibit both ureases competitively placed similarly in the active site and their phenyl rings are symmetrically placed between two positively charged nickel metal ions but their hydrogen bonding and hydrophobic interaction are varied with each ligand. Because of strong coordinative covalent bond in the metal centre, hydrogen bonding and hydrophobic contacts of these residues of the receptor with ligandcan be assigned as competitive inhibitors of both ureases that are also in agreement with steady-state inhibition kinetics and UV- visible spectroscopic data. Molecular modeling studies also revealed that upon the binding of biscoumarins, the geometry and coordination number of the Ni ions not change significantly. The results of molecular docking are shown in Figures 3, 4, 5, 6, 7, and 8.

### 3.2. Compound **3**-Urease Complex

Urease complex is stabilized only through hydrogen bonding and hydrophobic contacts with the residues (Arg 369 (2.66 Å) and Gly 368 (3.02 Å) while the hydrophobic contacts with the residues Met 318 and Leu 365 only). In case of compound** 3** there is no contact with the nickle metal centre and that is the reason showing pure classical noncompetitive type of inhibition. Compound** 3** is unable to reach the nickle metal centre; this may be because of the hindrance provided by methyl substituent at (R) position and ligands possibly unable to adopt the proper shape to enter the narrow active site of urease.

### 3.3. Compound **2**-Urease Complex

Steady-state kinetics data analysis suggested uncompetitive type of inhibition, docking also gave the same results. Principal interactions experienced by the ligand** 2** are hydrogen bonding and hydrophobic contacts, especially with the (Cys 322 (2.51 Å)) also exert hydrophobic interactions with (Glu 166, Lys 169, Ala 170, Met 367, Leu 365, and Ala 366). These are the major interaction which stabilized ligand** 2** in the receptor.

**Figure 9:**
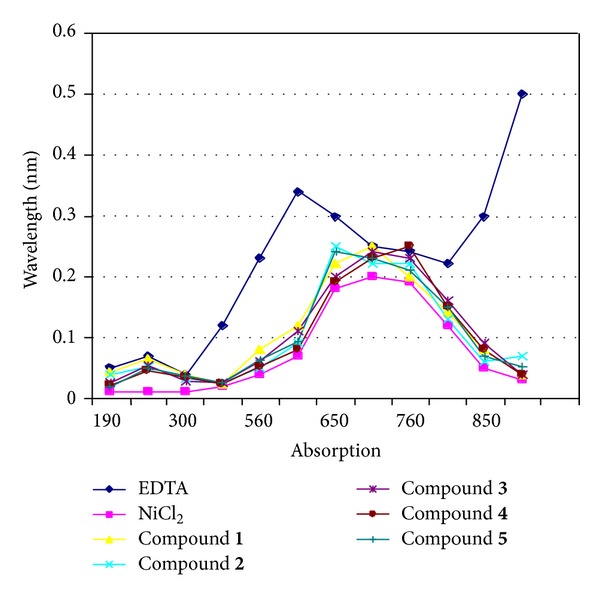
Absence of effect on NiCl_2_ spectrum of the ten selected Ligands compared with that of EDTA. The spectrum of NiCl_2_ (0.1 M) solution was followed between 190 and 900 nm at room temperature in absence or in presence of** 1**–**5** Ligands. *x*-axis absorption and *y*-axis wavelength (nm).

**Figure 10:**
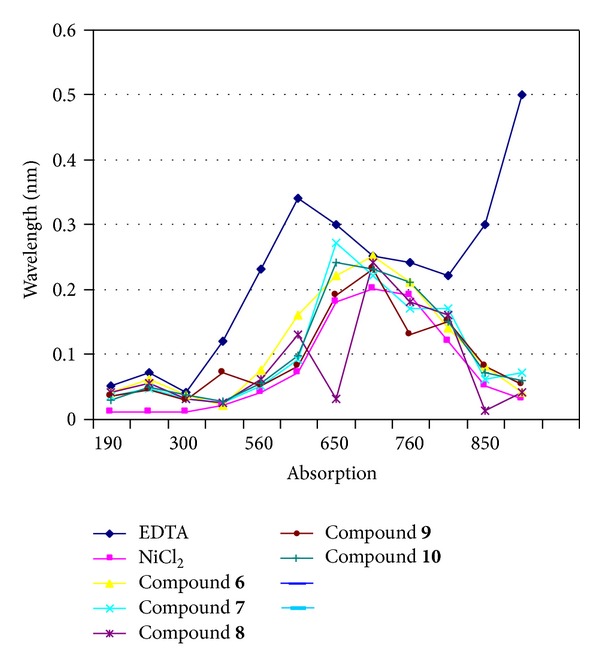
Absence of effect on NiCl_2_ spectrum of the ten selected Ligands compared with that of EDTA. The spectrum of NiCl_2_ (0.1 M) solution was followed between 190 and 900 nm at room temperature in absence or in presence of** 5**–**10** Ligands. *x*-axis absorption and *y*-axis wavelength (nm).

**Figure 11:**
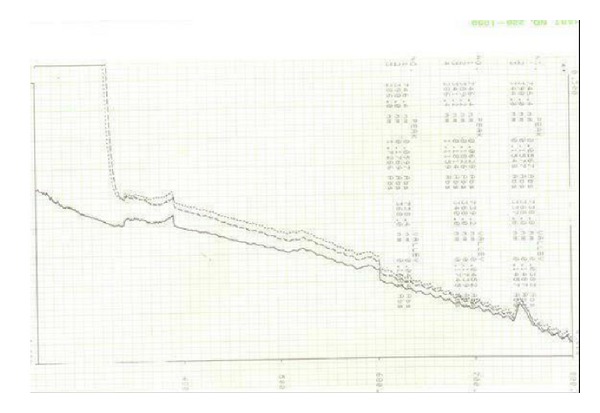
UV spectra of urease in the absence and presence of 15 and 30 *μ*M Ligand** 7**. Spectra of urease (56 mg/mL) in 10 mM EDTA, 100 mM K_2_PO_4_ Buffer pH 6.8 at 25°C.

**Figure 12:**
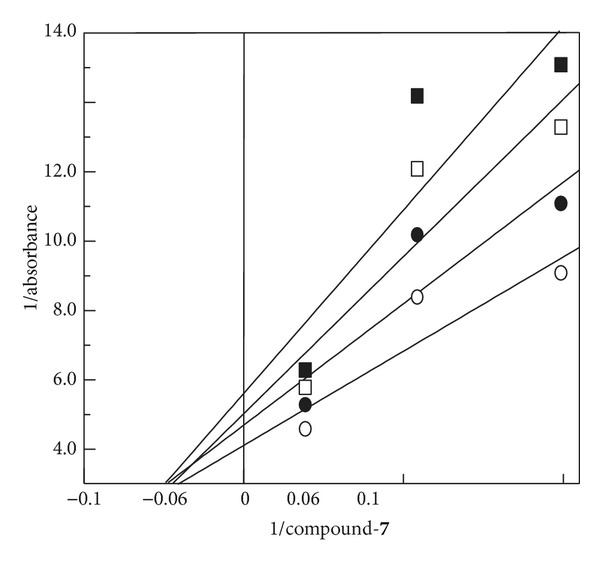
Double reciprocal plot of 1/absorbance versus 1/compound** 7**. The inverse change in absorbance at 25°C was plotted as a function of 1/compound** 7** at (■) 746 nm, (□) 390 nm, (●) 342 nm, and (○) 276 nm.

**Figure 13:**
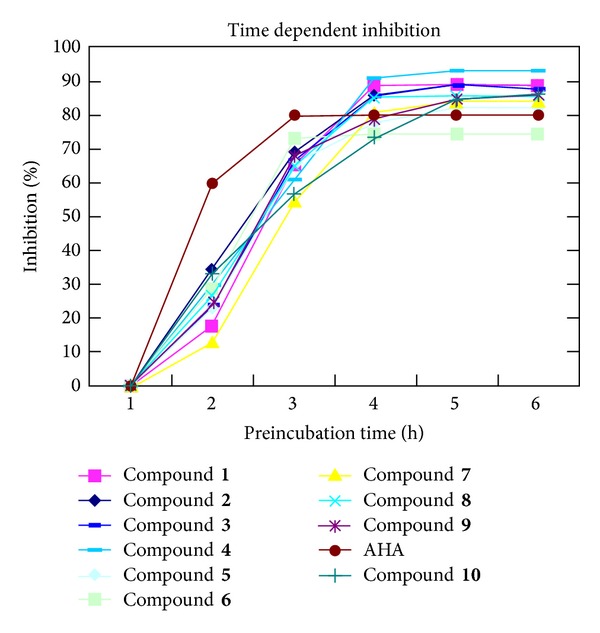
Time course of inhibition of jack bean urease activity by acetohydroxamic acid (AHA) and** 1**–**10** biscoumarins.

As we know that urease requires the presence of nickel ions in its active site for catalysis (EDTA inhibits the activity of the enzyme activity at high concentration), we have determined whether both inhibitory biscoumarin** 1**–**10** could have a chelating effect on nickel ions. Spectra comparing the effects of all ligands on the absorbance of a solution of nickel chloride were analyzed from 190 to 900 nm. While the addition of EDTA shifted the nickel ion spectrum to a lower wave length, from 700 nm to 620 nm, addition of the biscoumarin** 1**–**10** also modify the optical properties of the nickel-ion solution (Figures 9 and 10), indicating that all ligands chelates the nickel ions. These studies were also consistent with enzyme kinetics and UV-spectroscopic studies in case of J.B. urease but some discrepancies were found in case of* Bacillus* B.P. urease although compounds** 2**,** 3**,** 4**,** 5**, and** 9** also shows chelation with simple nickel but unable to make coordination when nickel is in protein and this may be because of the reason that nickel is not free in protein but surrounding by hundreds of amino acids which probably hindered these ligands to interact with nickel in the protein which is clear by our molecular docking results. UV-visible spectroscopy showed that all those biscoumarins which are competitive inhibitors of the urease bind to the nickel metallocenter. Indeed, the native enzyme has only a weak UV-visible spectrum, while the addition of biscoumarin compounds leads to spectroscopically detectable biscoumarin which induces the bridging of the two nickel ions in the active site and in case of all competitive inhibitors absorbance at 286 nm region is increased a lot which clearly shows that benzene ring is symmetrically sandwiched between the two nickel ions.

In order to evaluate the toxicity effects of compounds** 1**–**10** against human neutrophils, a standard operational protocol with acetohydroxamic acid as positive control was used. Acetohydroxamic acid is a urease inhibitor and is used as a drug for the treatment of urease associated diseases. The viability of human neutrphils (1 × 10^7^ cells/mL) in presence of 50 *μ*g/mL of compounds** 1**–**10** has been presented in Table 2. From the results it is clear that these compounds have safe profile in human neutrophils viability assay as that of acetohydroxamic acid.

Several classes of molecule have been tested as urease inhibitors both in medicine and in agriculture [4, 21]. The efficiency of the presently available inhibitors is low, however, and negative side effects on humans [1, 4, 21] and on the environment [22] have been reported. Therefore the potent urease inhibitory potential and safe profile against human neutrophils cell and plants (results of phytotoxicity are given in Table 3) make these compounds** 1**–**10** the possible therapeutic candidates for urease associated pathologies. Therefore there is an urgent need to bring these under study compounds in commercial use. The present study also provide important clues for drug design of more potent urease inhibitor based on the biscoumarin functionality.

## 4. Conclusion

We assess the enzyme kinetics, UV-visible spectroscopy, and molecular docking results in terms of the known protein structure of the enzyme and evaluate possible molecular interpretations for the site of biscoumarin binding and found that phenyl ring is the major active pharmacophore. The excellent in vitro potency and selectivity profile of the several compound described combined with their safe profile against human cell and plants suggests that these compounds may represent a viable lead series in the discovery of the new therapies for the treatment of urease associated problems. Present studies will be very helpful to understand the underlying mechanism of receptor-drug interaction. These mechanistic studies of the biscourmarin derivatives are expected to provide rational information for the design of new potential inhibitors of urease. In the process of exploring these biscourmarines, it was discovered that phenyl ring region plays very important role in inhibition in most cases; therefore exploitation of this region could possibly result in more potent selective inhibitors of urease.

## Figures and Tables

**Table 1:** 

Enzymes	Compounds	K_i_ (µM) ± SEM	K_m_ (mM)	K_mapp_ (mM)	V_max _ (µmol/min)^−1^	V_maxapp_	Type of inhibition
Urease (JB)	**1 **	19.3 ± 0.1	2.5	5.6	105	109	Competitive
	**2 **	71.0 ± 0.3	2.5	7.7	105	106	Competitive
	**3**	59.4 ± 0.1	2.5	8.3	105	108	Competitive
	**4**	65.9 ± 0.0	2.5	9.1	105	105	Competitive
	**5**	53.3 ± 0.4	2.5	7.8	105	103	Competitive
	**6**	75.0 ± 0.1	2.5	9.5	105	106	Competitive
	**7**	15.0 ± 0.1	2.5	7.6	105	102	Competitive
	**8**	21.5 ± 0.0	2.5	7.5	105	104	Competitive
	**9**	62.9 ± 0.5	2.5	11.6	105	108	Competitive
	**10**	68.0 ± 0.1	2.5	10.6	105	105	Competitive

Urease (BP)	**1**	15.5 ± 0.2	5.1	11.3	160	159.3	Competitive
	**2**	51.3 ± 0.4	5.1	5.2	160	98.0	Noncompetitive
	**3**	53.0 ± 0.01	5.1	4.0	160	115.3	Uncompetitive
	**4**	48.3 ± 0.01	5.1	3.8	160	101.0	Uncompetitive
	**5**	68.1 ± 0.3	5.1	3.9	160	93.7	Uncompetitive
	**6**	27.5 ± 0.0	5.1	13.0	160	159.2	Competitive
	**7**	13.3 ± 0.2	5.1	9.9	160	162.5	Competitive
	**8**	19.0 ± 0.1	5.1	7.7	160	161.7	Competitive
	**9**	59.5 ± 0.2	5.1	3.2	160	97.2	Uncompetitive
	**10**	63.6 ± 0.2	5.1	10.3	160	115.5	Competitive

*K*_*i*_ (dissociation constant or inhibition constant) was determined from nonlinear regression analysis by Dixon plot and secondary Lineweaver-Burk plot at various concentrations of **1–10**, *K*_*m*_ (Michaelis-Menten constant) is equal to the reciprocal of *x*-axis intersection, *V*_max⁡_ (maximal velocity) is equal to the reciprocal of *y*-axis intersection of each line for each concentration of **1–10** in the Lineweaver-Burk plot. The *V*_maxapp_ is equal to the reciprocal of *y*-axis intersection of each line for each concentration of **1–10** in Dixon plot (Each point in Lineweaver-Burk and represents the mean of three determinations). Urease (BP) (*Bacillus pasteurii* ureases) and urease (JB) (*Jack bean* urease).

**Table 2:** Results of Lemna Welv. phytotoxicity assay.

Compound	Conc. of compound (*μ*g/mL)		
	1000	100	10
**1**	80	60.3	9.9
**2**	52.7	33	6.5
**3**	54.8	22.5	6.4
**4**	100	100	38.23
**5**	61.4	25.00	9.00
**6**	69.6	42.50	10.60
**7**	78	20.20	7.50
**8**	70.0	22.99	13.00
**9**	100	50.00	41.66
**10**	57.14	47.6	23.8

**Table 3:** Viability of human neutrophils (1 × 10^7^ cells/mL) in the presence of biscoumarins **1–10**.

Compounds	Conc. *µ*g/mL	Viability [%]
**1**	200	67.54 ± 3.1
**2 **	200	85.07 ± 2.5
**3**	200	100.0 ± 1.0
**4**	200	13.21 ± 4.2
**5**	200	100.0 ± 0.5
**6**	200	45.54 ± 4.0
**7**	200	100.0 ± 4.3
**8**	200	100.0 ± 5.2
**9**	200	100.0 ± 5.2
**10**	200	95.4 ± 5.2
